# Antidotes

**Published:** 1844-10

**Authors:** 


*Antidotes.*—Messrs. Bouchardat and Sandras, from their experiments, consigned in the Bulletin General de Therapeutique, conclude that the antidotes *to corrosive sublimate* are a mixture of the powder of zinc and iron; the persulphuret of the hydrated peroxide of iron; *to copper*, powers of zinc and iron mixed; porphyrised iron: zinc filings; persulphuret of hydrated peroxide of iron; *to lead*, persulphuret of hydrated peroxide of iron; *to arsenious acid*, humid and dry hydrated peroxide of iron; humid persulphuret of hydrated peroxide of iron. This last named substance may be administered in cases where the exact nature of the poisons is not known. As to the *modus administrandi;* the powders of zinc and iron may be given in an electuary, and the peroxide of iron, and the persulphuret, in the form of a jelly, in which they may be kept in the apothecary's shop. The doses—ʒij. of the powders of zinc and iron are sufficient for ℈j. of acetate of copper; ℥ij. of the magma of the persulphuret for ℈j. of acetate of copper, and grs. vj. of arsenious acid; ℥iv. of the magma of the humid hydrated peroxide of iron, or ʒxxj. of dry hydrated peroxide of iron for grs. vj. of arsenious acid. Several glasses of tepid water must be given soon after their administration, and other means employed to produce vomiting. The sooner the antidote is given the greater the chance for sucess; from the effects of the acetate of copper the patient may recover, even should forty minutes elapse before its administration; but arsenious acid is dissolved more rapidly.—*Braithwaite's Retrospect—from Med. Times.*

